# Preparation and Evaluation of Nano-vesicles of Brimonidine Tartrate as an Ocular Drug Delivery System

**DOI:** 10.4103/0975-1483.71623

**Published:** 2010

**Authors:** P Prabhu, Kumar R Nitish, M Koland, NM Harish, K Vijayanarayan, G Dhondge, RN Charyulu

**Affiliations:** *Department of Pharmaceutics, N.G.S.M. Institute of Pharmaceutical Sciences, Paneer, Deralakatte, Mangalore-575 018, India*

**Keywords:** Brimonidine tartrate, film hydration, intraocular presuure, nano size, niosome, liposome

## Abstract

The objective of the present investigation was to design a vesicular formulation of brimonidine tartrate and evaluate its ability to reduce the dosing frequency and improve the therapeutic efficacy of the drug. Nano-vesicles of brimonidine tartrate were prepared by film hydration method. The prepared vesicles were evaluated for photomicroscopic characteristics, entrapment efficiency, *in vitro*, and *ex-in vitro* drug release and *in vivo* intraocular pressure (IOP) lowering activity. The methods employed for preparation of vesicles produced nano vesicles of acceptable shape and size. The *in vitro*, and *ex-in vitro* drug release studies showed that there was slow and prolonged release of the drug, which followed zero-order kinetics. The IOP-lowering activity of nano vesicles was determined and compared with that of pure drug solution and showed that the IOP-lowering action of nano-vesicles sustained for a longer period of time. Stability studies revealed that the vesicle formulations were stable at the temperature range of 2-8°C, with no change in shape and drug content. The results of the study indicate that it is possible to develop a safe and physiologically effective topical formulation that is also convenient for patients.

## INTRODUCTION

Vesicular drug delivery system using colloidal particulate carriers (liposomes or niosomes) have distinct advantages over conventional dosage forms because colloidal particles can act as drug containing reservoirs. Modification of the particle composition or surface can adjust the affinity for the target site and/or the drug release rate. The slow drug release from the carrier system may reduce the toxicity of the drug and hence these carriers play an important role in drug delivery.[1] The primary objective of creating new drug delivery system in ocular therapeutics is to improve existing ocular dosage forms and exploit newer drug delivery systems for improving the bioavailability of an existing drug molecule. Eye drops are the most preferred method of administering drugs to the eye. Although topical and localized applications are still an acceptable and preferred route for ocular drug delivery, such dosage forms are no longer sufficient to combat ocular diseases such as glaucoma as they have poor bioavailability, which is the result of the efficient mechanisms protecting the eye from harmful materials and agents. Such protective mechanisms include reflex blinking, lacrimation, tear turnover, and drainage of tear, all of which results in the rapid removal of the drug from the eye surface. Also frequent instillation of concentrated medication is required at the site of action and this is inconvenient for the patient.[2] The development of various vesicular drug delivery systems allows the entrapment of the drug molecule into a lipid bilayer or in surfactant vesicles and thus allows us to increase drug concentration at the site of application and, thus, to improve bioavailability. Such vesicles (liposomes and niosomes) act as carriers for controlled ocular drug delivery by preventing metabolism of the drug by enzymes present at the corneal epithelial surface. Vesicle-entrapped drug can be easily administered in liquid dosage forms such as eye drops with good patient compliance, modulated drug release profile and high drug pay-load. Brimonidine tartrate is an α_2_-adrenergic receptor agonist. It acts by decreasing synthesis of aqueous humor, as well as by increasing its drainage from the eye. As a treatment for glaucoma, it is usually given in a 0.2% w/v (2 mg/mL) eye drop form that has to be administered 2-3 times daily. In humans, after topical dosing, the mean apparent half-life of brimonidine tartrate in the systemic circulation is approximately 3 h. The plasma protein binding of brimonidine tartrate after topical dosing in humans is approximately 29%. The major part of the dose (around 75% of the dose) is excreted within 5 days as metabolites in urine. The drug is used to treat open-angle glaucoma or ocular hypertension and is also used to induce miosis in people suffering from poor night vision after Lasik or PRK surgery.[3–5] In the present study, to produce a sustained effect of the drug, an attempt was made to develop a vesicular drug delivery system (liposomes and niosomes) of brimonidine tartrate for ocular administration. We investigated its intraocular pressure (IOP) lowering activity and its other physical properties and the drug release pattern.

## MATERIALS AND METHODS

### Materials

Brimonidine tartrate was obtained from FDC Aurangabad, India, as a gift sample. Cholesterol, soya lecithin, and span-60 were obtained from CDH Laboratories, New Delhi. Diethyl ether, chloroform, alcohol, alpha-tocopherol, potassium dihydrogen phosphate, and disodium hydrogen phosphate were obtained from E-Merck India Ltd, Mumbai. DPPC (1, 2–Dipalmitoyl-sn-glycero-3-phosphocholine) was obtained as a gift sample from Genzyme Pharmaceuticals, Switzerland.

### Preparation of vesicles

In the present study, three formulations each of liposomes and niosomes, of brimonidine tartrate were prepared by film hydration method.[6] All the lipid components (including surfactant Span-60, for niosomes) of the formulation (as per the formula given in Table 1) were taken in a round-bottom flask and dissolved in sufficient quantity (10 mL) of organic solvent (chloroform). The organic solvent was evaporated using a rotary flash evaporator under reduced pressure at a temperature of about 60°C till a lipid film was formed inside the flask. The dried lipid film obtained was hydrated with aqueous phase of phosphate buffer (pH 7.4, 10 mL) containing the drug. The flask was shaken for 1 h to get liposomal or niosomal formulations. It was allowed to equilibrate at room temperature. Liposomal formulations were named as LF1, LF2, and LF3, and the niosomal formulations were named NF1, NF2 and NF3. The stable colloidal suspension was then sonicated for two cycles. During the first cycle, the suspension was sonicated at 80% amplitude with a pulse of 0.5 cycles per second for a period of 3 min, followed by 3 min rest (excess heat may be generated during probe sonication, which may damage the lipids). After 3 min, the second cycle was processed for 3 min at 80% amplitude with 0.5 cycles per sec pulse for another 3 min. The size of the vesicles was analyzed by Nano Zeta-sizer (Malvern, Instruments Ltd, USA) after sonication.

**Table 1:** Composition of formulations and their characterization

Formulation code	Formulation ratio (drug: cholesterol: DPCC/span 60)	Average particle size (µ)	Percentage drug entrapment efficiency
LF 1	1:1:1	6.5	32.34
LF 2	1:1:2	6.7	39.78
LF 3	1:1:3	7.2	42.43
NF1	1:1:1	6.7	32.00
NF2	1:1:2	7.2	41.20
NF 3	1:1:3	7.4	43.20

### Determination of size and shape

Particle size, and the polydispersity index of the vesicles were determined using Malvern Zeta sizer.

### Entrapment efficiency

Entrapment efficiency of brimonidine tartrate in the vesicles was determined as follows: After sonication, 1 mL of the vesicle suspension was taken in a 1 mL microcentrifuge tube, and centrifuged at 20,000 rpm for 1 h at 4°C in a cold centrifuge to get a white pellet. To the pellet, 500 µL of 0.1 N NaOH (the drug is highly soluble in 0.1N NaOH) was added and vortexed thoroughly for 3 min to get a white suspension. To this, 5 mL methanol was added to get a clear solution. This was further vortexed for 2 min to ensure that the vesicles were lysed completely to release the drug.[7] This solution (1 mL) was further diluted with methanol and the absorbance was determined using a Shimadzu UV/VIS spectrophotometer.

The entrapment efficiency (EE) was calculated using the following formula:

$$\mathrm{Percentage}\;\mathrm{entrapment}\;\left(\%\mathrm{EE}\right)\;=\;\frac{\mathrm{Entrapped}\;\mathrm{drug}\;\left(\mathrm{mg}/\mathrm{mL}\right)}{\mathrm{Total}\;\mathrm{drug}\;\mathrm{added}\;\left(\mathrm{mg}/\mathrm{mL}\right)}\;\times \;100$$

### *In vitro* drug release study

*In vitro* drug release study for formulations of vesicles was studied by membrane diffusion technique.[8] The diffusion medium was 19 mL of freshly prepared glutathione bicarbonated ringer (GBR) equilibrated at 37±0.5°C temperature. The pH of the medium was maintained at 7.2-7.4 by passing CO_2_. This medium closely resembles simulated tear fluid (STF). The samples were analyzed spectrophotometrically for concentration of brimonidine tartrate at 320 nm. The experimental data was subjected to statistical analysis using one-way ANOVA. *P*< 0.05, was considered statistically significant.

### *Ex- in vitro* drug release study

*ex-in vitro* drug release study for prepared vesicular formulations was studied by the method described above. In this study, porcine cornea was used as the diffusion membrane. All the procedures followed were similar to that explained under *in vitro* drug release study.

### *In vivo* intraocular pressure lowering activity

*In vivo* IOP lowering activity of vesicular preparations of brimonidine tartrate was studied in normotensive male albino rabbits weighing 1.2-2.5 kg. The animals were housed under well controlled conditions of temperature (22±2°C), and humidity (55±5%), with a 2/12-h light-dark cycle, and free access to food and water. The rabbits were divided into three groups containing 6 rabbits each (6 each for marketed product, niosomes, and liposomesrespectively). Group 1, was treated with marketed formulation (Alphagan^®^ eye drops, 0.02%), group 2, was treated with liposomes, and group 3, was treated with niosomes. The protocol of the experiment was approved by the Institutional Animal Ethics Committee (K.S. Hegde Medical Academy, Reg. No 115/1999/CPCSEA).

To induce acute glaucoma, 5% dextrose solution (15 mL/kg) was intravenously infused through the marginal ear vein. The basal intraocular pressure was measured by with tonometere. The drug formulations (20 µL) were administered to the rabbits 30 min before the administration of dextrose solution. The IOP changes were recorded every 30 min, till the pressure difference between the control eye and treated eye was found to be zero. The formulation was instilled on to the corneal surface of one eye, and with the contra-lateral eye being the control. IOP was measured by the tonometry method with the help of a Schiotz tonometere. All IOP measurements were carried out by the same operator, and using same tonometere. Each rabbit was given a washout period of 3 days after every treatment. The ocular hypotensive activity was expressed as the average difference in IOP between the treated and control eye of the same rabbit, according to the equation Δ IOP = IOP of treated eye — IOP of control eye.[8,9] The experimental data was subjected to statistical analysis, using one-way ANOVA. *P*< 0.05, was considered to be statistically significant.

### Stability study

In the present work, a stability study was carried out for selected formulations LF3 and NF3. They were stored at room temperature and under refrigeration (2-8°C), for 8 weeks, and the formulations were then evaluated for the drug content and shape of the vesicles.

### Determination of drug release kinetics

To know the mechanism of drug release from these formulations, the data were treated according to first-order (log cumulative percentage of drug remaining *vs* time), Higuchi’s (cumulative percentage of drug released *vs* square root of time), and zero-order (cumulative amount of drug released *vs* time) pattern.[10]

## RESULTS AND DISCUSSION

**Figure 1:**
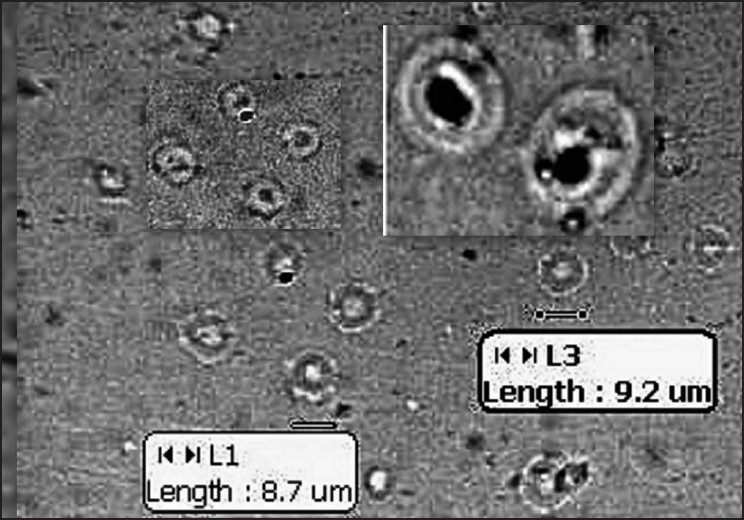
Photomicrograph of liposomes before sonication

**Figure 2:**
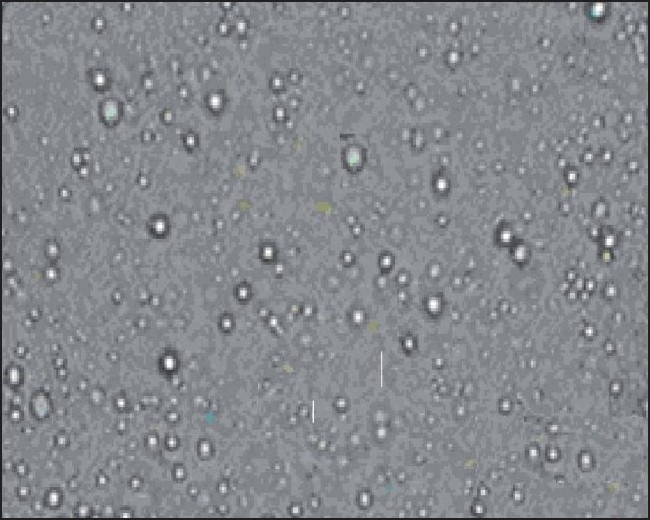
Photomicrograph of niosomes before sonication

Vesicular formulations were prepared using the film hydration method and were characterized [Table 1]. The average size of the vesicles ranged from 6.50 µ-7.40 µ, before sonication and all the vesicles were found to be spherical in shape and multilamellar [Figures 1 and 2]. The sonication resulted in much smaller vesicles, which is essential if irritation to the eye is to be avoided. The size of particles in ophthalmic dosage forms, apart from influencing bioavailability, plays an important role in its irritation potential; hence, it is recommended that particles of ophthalmic solution be less than 10 µ so as to minimize irritation to the eye.[8] The size of sonicated vesicles was found to be in the range of 210-245 nm. The amount of drug entrapped in vesicles ranged between 32% and 42% w/w [Table 1].

The comparison of the *in vitro* drug release profiles of pure drug solution and for each formulation is summarized in Table 2. It was observed that pure drug solution released approximately 75% of drug within 2 h, while vesicle formulations LF3 and NF3 showed 18% and 22% drug release respectively in 8 h. Liposomes showed marginal increment in drug release with increase in the lipid composition, i.e., at drug: cholesterol: DPCC ratio of 1:1:3, the release was found to be faster compared to that at the ratio of 1:1:1 (22.15% *vs* 18.12%). The release of drug from niosomes was found to be significantly (*P*>.05) different from that of liposomes. For example NF1 released 24% of drug compared to 18% of LF1. The result of *in vitro* drug release profiles of the formulations showed that vesicular formulations provides the more prolonged release of drug when compared to pure drug solution.

**Table 2:** Comparative *in-vitro* dissolution profile of different formulations

Time (h)	Percentage amount of drug release						
	Pure drug solution	Formulation					
		NF1	NF2	NF3	LF1	LF2	LF3
1	68.00 ± 1.4	06.43 ± 0.79	10.69 ± 0.99	14.13 ± 0.99	03.26 ± 0.45	04.46 ± 0.99	05.81 ± 0.99
2	77.40 ± 1.2	13.67 ± 0.68	19.29 ± 0.98	22.35 ± 1.09	10.86 ± 0.37	12.86 ± 0.98	12.75 ± 0.98
3	78.80 ± 1.3	16.43 ± 0.93	20.28 ± 0.97	24.91 ± 1.16	13.42 ± 0.56	14.40 ± 0.99	13.76 ± 1.00
4	78.30 ± 1.2	17.18 ± 0.77	21.42 ± 0.94	26.52 ± 0.98	14.38 ± 0.91	14.58 ± 0.98	16.55 ± 1.10
5	80.00 ± 1.3	21.64 ± 0.94	22.69 ± 0.99	27.99 ± 0.98	15.67 ± 0.35	16.67 ± 0.97	19.05 ± 0.99
6	79.64 ± 1.4	22.13 ± 0.95	23.19 ± 0.98	29.30 ± 0.99	16.23 ± 0.45	16.83 ± 0.98	19.40 ± 0.98
7	81.54 ± 1.6	23.58 ± 0.87	24.19 ± 0.99	31.11 ± 0.97	16.92 ± 1.12	16.98 ± 1.00	21.11 ± 0.99
8	83.42 ± 1.1	24.12 ± 1.12	27.19 ± 1.05	33.85 ± 0.98	18.12 ± 1.23	18.92 ± 1.10	22.51 ± 0.98

The comparative *ex-in vitro* drug release profile for pure drug solution and vesicular formulations is summarized in Table 3. The *in vitro* and *ex-in vitro* release models did not show any significant difference in drug release due to the effect of the diffusion membrane. The prolonged release rate may be attributed largely to the drug transport by a diffusion controlled mechanism from vesicles. The *in vitro* and *ex-in vitro* drug release studies showed that, there was slow and prolonged release of drug from all the formulations following zero-order kinetics.

**Table 3:** Comparative *ex-in vitro* dissolution profile of different formulations

Time (h)	Percentage amount of drug release						
	Pure drug solution	Formulation					
		NF1	NF2	NF3	LF1	LF2	LF3
1	67.56±1.60	07.89 ± 0.99	06.37 ± 0.99	12.89 ± 0.99	6.81±0.89	7.89 ± 0.99	05.37 ± 0.93
2	78.31±1.10	13.04 ± 0.98	13.12 ± 0.98	15.68 ± 0.98	12.12 ± 0.45	13.04 ± 0.98	11.13 ± 0.93
3	78.80±1.40	13.36 ± 0.99	14.31 ± 1.09	18.92 ± 0.97	13.17 ± 0.67	13.36 ± 0.99	13.21 ± 1.10
4	78.98±1.30	14.00 ± 0.98	15.87 ± 1.17	24.76 ± 0.94	13.98 ± 0.28	14.00 ± 0.98	14.87 ± 0.98
5	80.00±1.30	15.64 ± 0.97	16.59 ± 0.99	27.56 ± 0.99	14.69 ± 0.57	15.64 ± 0.97	15.52 ± 0.56
6	80.64±1.25	17.56 ± 0.99	18.57 ± 0.98	27.89 ± 0.98	16.34 ± 0.78	17.56 ± 0.99	17.53 ± 0.98
7	81.74±1.80	17.76 ± 1.05	20.55 ± 0.99	28.56 ± 0.99	17.26 ± 0.98	17.76 ± 1.00	19.38 ± 0.78
8	83.12±1.30	18.89 ± 1.10	22.56 ± 0.98	29.81 ± 1.07	18.39 ± 1.05	18.89 ± 1.10	21.54 ± 0.98

**Figure 3:**
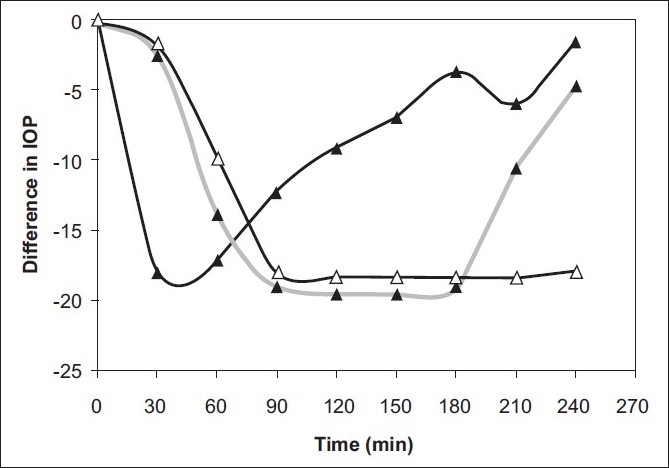
Comparative IOP-lowering activity. -Δ-Niosomal formulation. -▲-Pure drug solution -▲- Liposomal formulation

To study the *in vivo* performance of prepared formulations, IOP lowering activity was determined. It was found that with vesicular formulations IOP lowering activity was sustained for longer periods (3-4 h). The marketed product showed activity within 30 min, but the activity not sustained beyond 60 min. It was found that the IOP difference produced between pure drug solution and vesicles is very significant (*P*> 0.05). Vesicular formulations sustained the action for a prolonged period of time (240 min) [Figure 3]. The IOP-lowering activity of pure drug solution did not sustain long. It was also observed that at the end of 240 min, the effect of all the formulations was found to be nil. This may have been because rise in IOP induced by injection of 5% dextrose solution does not last long. Nevertheless, the experimental data proves the sustained action of vesicular formulation in comparison to marketed eye drops. The better reduction in IOP with vesicles may probably be due to the better partitioning of drug between vesicle and eye corneal surface. Also, the release of drug from vesicles increases the concentration at the corneal surface, and thus longer contact time of vesicles at the corneal surface, leads to higher bioavailability of drug. Thus, the vesicles act as a drug carrier, and change the rate and extent of absorption, resulting in reduction of IOP for more prolonged periods.

The time to onset of action, duration of action, percentage IOP-lowering activity, and peak effective time were also measured. The maximum IOP (IOP of contra-lateral eye, 30.4 mmHg) was recorded between 1.5-2.5 h and this was considered as 100% glaucoma induction. During the same period, the reduction in IOP observed in eye treated with formulation considered as percentage reduction in IOP. The marketed formulation produced the maximum percentage reduction of 59% between 1.0-1.5 h. The vesicles showed a maximum of 39% reduction in IOP between 2-2.5 h. Though, a higher percentage reduction of IOP was observed with the marketed product, the effect was not sustained. In comparison, vesicular formulations produced a IOP-lowering action that was sustained longer duration of time (3.5-4 h), [Table 4].

**Table 4:** Change in IOP-lowering activity parameters

Formulation	Onset of time (h)	Peak effective time (h)	Duration of sustainability of action	% IOP-lowering activity
Marketed formulation	0.5±0.2	1.5±0.2	0.5±0.4	59
LF3	0.5±0.4	1.1±0.3	3.0±0.5	39
NF3	0.5±0.2	1.8±0.2	4.0±0.9	39

The result of the stability study showed satisfactory and acceptable stability with the vesicles stored at temperatures of (2-8°C). However, liposomes stored at room temperature showed sign of drug leak and structural deformation, niosomes stored at room temperature showed acceptable stability, with no change in shape and no significant difference in drug content.

## CONCLUSION

The *in vitro* and *ex-in vitro* drug release studies showed sustained release zero-order kinetics. The *in vivo* IOP-lowering activity of vesicular formulations was found to be significant and sustained, which is promisimg for its physiological effectiveness. Thus, vesicular systems have the potential to meet the need for an ophthalmic drug delivery system that not only has the convenience of a drop formulation, but, can also localize and maintain drug activity at the site for a long period of time. This would help to reduce the frequency of drug administration and thereby improve patient compliance.
